# High yielding ability of a large-grain rice cultivar, Akita 63

**DOI:** 10.1038/s41598-020-69289-0

**Published:** 2020-07-22

**Authors:** Amane Makino, Yoshihiro Kaneta, Mitsuhiro Obara, Keiki Ishiyama, Keiichi Kanno, Eri Kondo, Yuji Suzuki, Tadahiko Mae

**Affiliations:** 1 Graduate School of Agricultural Science, Tohoku University, 468-1 Aramaki-Aoba, Aoba-ku, Sendai, 980-8572 Japan; 2 Faculty of Bioresource Sciences, Akita Prefectural University, Shimosinjyou, Akita 010-0195 Japan; 3 Japan International Research Center for Agricultural Sciences, Tsukuba, Ibaraki 305-8686 Japan; 4 Present Address: Faculty of Agriculture, Iwate University, Morioka, 020-8550 Japan

**Keywords:** Ecology, Plant sciences

## Abstract

To increase the yield potential while limiting the environmental impact of N management practices is an important issue in rice cultivation. The large-grain rice cultivar Akita 63 showed higher N-use efficiency for grain production. To elucidate this, we analyzed yield characteristics of Akita 63 in comparison with those of a maternal cultivar, Oochikara with a large grain, a paternal cultivar, Akita 39 with a normal grain, and a Japanese leading cultivar, Akitakomachi. The yields of Akita 63 were 20% higher than those of Oochikara and Akita 39, and 50% higher than those of Akitakomachi for the same N application. Akita 63 showed superior N uptake capacity. Whereas a trade-off between single grain weight and grain number was found for Oochikara, Akita 63 did not show such a relationship. The success in Akita 63 breeding was due to overcoming such a trade-off. Akita 63 had the large-grain alleles of *GS3* and *qSW5*. Thus, an enlargement of grain size can have a great impact on an increase in yield with improved N-use efficiency. However, an enlargement of sink capacity led to source limitation. Thus, both sink and source improvements are essential for a further increase in the yield of today’s high-yielding cultivars.

## Introduction

Rice is the most important food crop in the world, reaching 782 million tons of food production in 2018, and surpassing the production of wheat according to FAOSTAT (https://faostat3.fao.org). However, more than 200 million tons of rice production is estimated to be additionally required to meet expanding demand within the next 30–40 years. This means that rice cultivars with higher yield potential must be developed because the scope for expansion of irrigated rice area is already limited.

Breeding of semi-dwarf rice varieties in the 1960s made a great contribution to increasing yield potential, which is called as the Green Revolution in Asia. Since the introduction of semi-dwarf traits into rice was able to solve the lodging problem and led to the development of a plant type with high light utilization, a large input of N fertilizer was feasible. Thus, a great increase in yield potential in semi-dwarf cultivars strongly depends on N application. On the other hand, large inputs of N fertilizer in turn have drawn much attention to the environmental impact of N application practices^1^.

After the success of semi-dwarf breeding, the main targets of rice improvement have moved to the introduction of disease and insect resistance, improvement in grain quality and shortened growth duration. Concerning yield potential, the focus has been on developing hybrid rice and new-plant-type rice with large panicle, low tillering and lodging resistance^2^. However, there has been no actual increase in the yield potential since the release of the first semi-dwarf cultivars^3^. The increase in yield up to now has been the result of dwarfing and the use of cheap N fertilizers and herbicides^4^.

Enlargement of single grain size has also had a great impact on the increase in yield potential because grain size as well as the number of grains is the major determinant of yield potential. In rice, since single grain size is genetically constant^5^, with respect to yield improvement, an increase in the number of grains has been given more attention^6^. Actually, semi-dwarf *indica* cultivars tend to have a relatively greater number of grains^7^. On the other hand, there have been few reports analyzing the effects of enlargement of grain size on the yield potential^8,9^. Meanwhile, we have found that a large-grain cultivar, Akita 63, showed high yield potential as a new type of high-yielding cultivar^10^. In Akita 63, the single grain weight was about 35% larger than normal and the yield was 20–60% greater than that of the reference cultivars for the same degree N application. Since this cultivar also showed high yield for a given amount of plant N, culture of Akita 63 can lead to a reduction of the environmental impact of N fertilizer. However, the Japanese large grained cultivar Oochikara, which is the maternal cultivar of Akita 63, did not necessarily produce high yields^9^. Thus, the effect of single grain weight on yield potential remains uncertain.

Several quantitative trait loci (QTLs) determining grain size have been fine-mapped. As genes determining enlarged grains, *GS3*^11^, *GW2*^12^, *TGW6*^13^, *qSW5*^14^, *GS5*^15^, *GW8*^16^ and *GL2/GS2*^17,18^ have been identified. Those reports have clarified various aspects of the mechanisms of the enlargement of grain size. Loss or reduction of function of *GS3*, *GW2, TGW6*, *qSW5* and *GL2/GS2* results in enlarged grains whereas higher expression of *GS5* and *GW8* is associated with large grains. In contrast to the gene which increases the number of grains, *Gn1a* was identified as the first QTL gene from a high-yielding *indica* cultivar^19^; however, the genes determining grain size are not necessarily observed for high-yielding cultivars. Thus, although single grain weight is one of the yield components, the causal relationship between genes controlling grain size and yield potential is still not known.

In the present study, we first examined the high-yielding abilities of Akita 63, in comparison with yields and yield components of a maternal cultivar, Oochikara, with large grains, a paternal cultivar, Akita 39, with normal grains, and a conventional cultivar, Akitakomachi, which is one of the leading cultivars in Japan at present. All cultivars were grown in a field with different levels of N application for 4 years and the yield and yield components were analyzed in relation to total N content of plant above ground. In addition, we elucidated if the large-grain alleles of *GS3*, *GW2*, *TGW6* and *qSW5* existed in those cultivars and contributed to grain size, and examined yield potential in relation to grain size.

## Results

**Figure 1:**
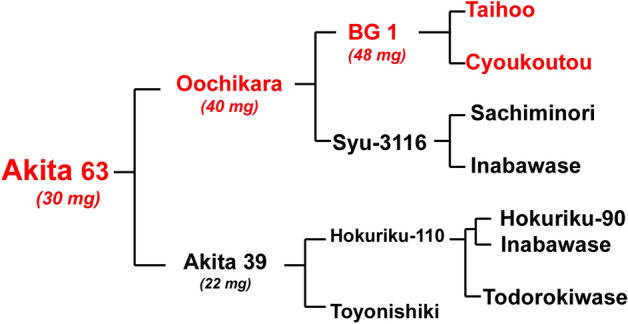
Genealogy of a large-grain rice cultivar, Akita 63. Red color indicates cultivars with large grains. The value in parenthesis shows the average weight of brown rice grain (see, Table 1; Fig. 2).

**Figure 2:**
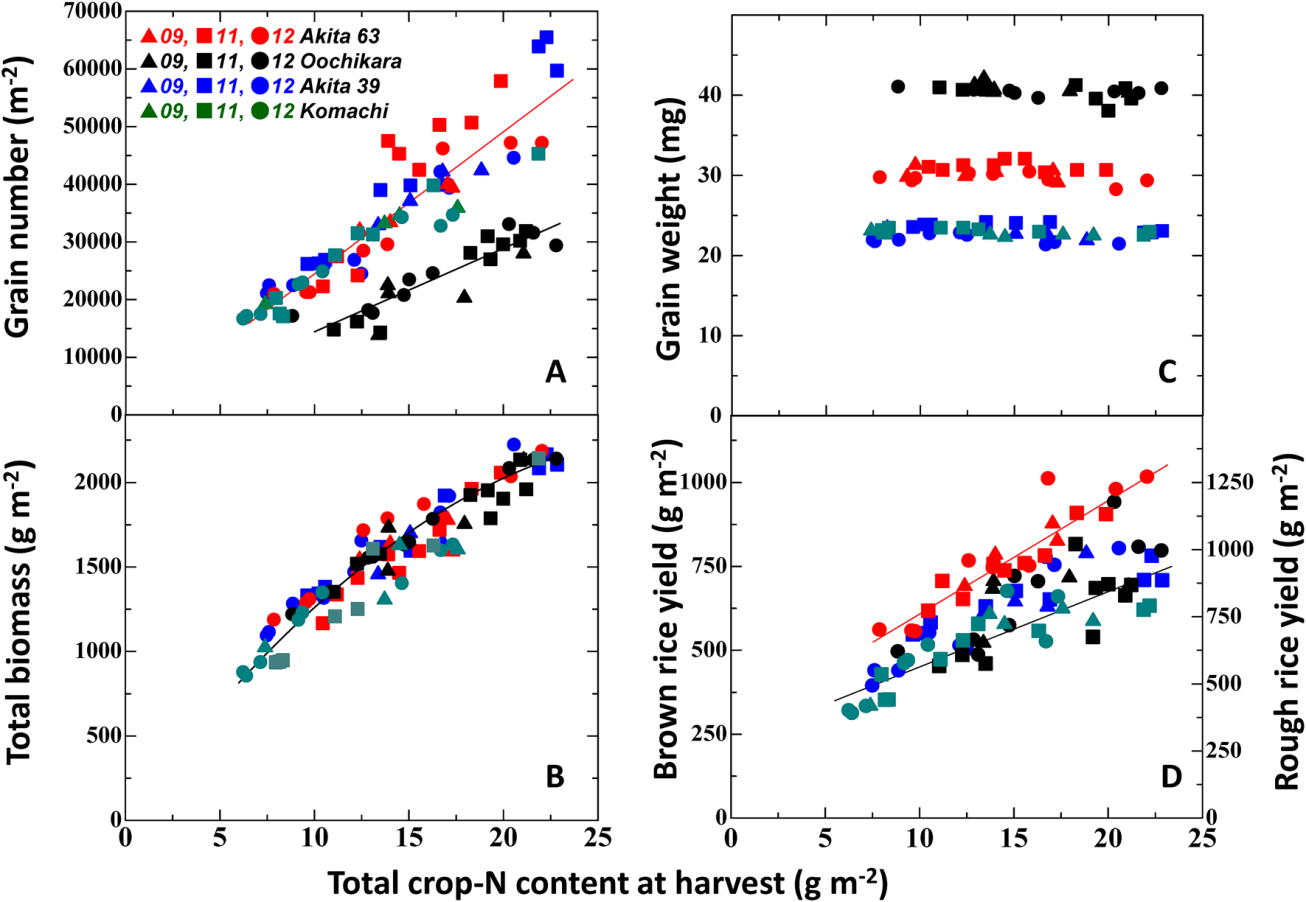
Number of grains, total aboveground biomass, single brown grain weight and yield versus total crop-N content of the above-ground section at harvest. Red symbols, Akita 63; black symbols, Oochikara; blue symbols, Akita39; green symbols, Akitakomachi. All data are taken from four cultivars grown in an experimental field of the Agricultural Experimental Station of Akita Prefecture in 2009 (triangles), 2011 (squares) and 2012 (circles). For number of grains (**A**), the red line represents the linear regression through the origin of the pooled dataset of Akita 63, Akita 39 and Akitakomachi, Y = 2460X, r = 0.917, P < 0.001; the black line represents the linear regression through the origin of the dataset of Oochikara, Y = 1420X, r = 0.898, P < 0.001. A significant difference in the regression was found between Oochikara and other three cultivars (see, Supplementary Tables S3, S4). For total aboveground biomass (**B**), the line represents the quadratic regression line through the origin of the pooled dataset of four varieties, Y = − 2.31X^2^ + 146X, r = 0.947 (Y = 106.7X, r = 0.858 for linear regression analysis). For brown rice yield (**D**), the red line represents the linear regression of the dataset of Akita 63, Y = 34.5X + 265, r = 0.929, P < 0.001; the black line represents the linear regression of the pooled dataset of Oochikara, Akita 39 and Akitakomachi Y = 24.0X + 238, r = 0.830, P < 0.001. A significant difference in the regression was found between Akita 63 and other three cultivars (see, Supplementary Tables S3, S4). The regression analysis data for grain number and yield are presented in Supplementary Tables S3. Significant differences (P value) in the slope and y-axis intercept of two different regression lines between cultivars were obtained by correction using the Bonferroni method to avoid family wise error (Supplementary Table S4).

Akita 63 was bred between Oochikara, which has large grains (ca. 40 mg per brown rice grain), as a maternal cultivar and Akita 39, which has normal grains (22 mg per brown rice grain), as a paternal cultivar (Fig. 1). The brown grain of present standard rice cultivars weighs about 22 mg (Table S1). Both Oochikara and Akita 39 have semi-dwarf status, which is derived from Inabawase. The traits of grain size of Oochikara were derived from those of BG-1, and BG-1 was obtained from a cross between Taihoo and Choukoutou^9^. Taihoo is a Japanese cultivar with wide grains while Choukoutou is a Chinese cultivar with long grains. Although Akita 63 has large grains (30 mg per brown rice grain), the single grain weight is a little smaller than those of Oochikara (40 mg per brown rice grain, Figs. 1, 2; Table 1).

**Table 1:** Yield (brown rice), yield components total biomass and total crop-N content of the above-ground section of Akita 63, Oochikara, Akita 39, and Akitakomachi.

N application (g N m^−2^)	Cultivar	Yield sieved brown rice (g m^−2^)		No. of grains ( × 10^3^ m^−2^)	Fertility (%)	Grain wt brown rice (mg)	Total biomass aboveground (g m^−2^)	Total N aboveground (g m^−2^)
13	Akita 63	928 ± 31^a^	(1,160)	45.4 ± 2.9^a^	69.0 ± 5.0^a^	30.0 ± 0.4^a^	1944 ± 63^a^	18.5 ± 0.7^a^
	Oochikara	791 ± 56^b^	(989)	28.6 ± 1.5^b^	73.8 ± 4.5^a^	40.0 ± 0.5^b^	2098 ± 120^a^	21.0 ± 0.4^a^
	Akita 39	770 ± 33^b^	(962)	47.7 ± 5.1^a^	75.1 ± 8.6^a^	22.3 ± 0.3^c^	1,890 ± 119^a^	19.8 ± 1.3^a^
	Akitakomachi	635 ± 25^c^	(794)	37.5 ± 2.0^ab^	77.8 ± 5.3^a^	22.5 ± 0.3^c^	1,730 ± 109^a^	18.0 ± 1.1^a^
6	Akita 63	780 ± 36^a^	(975)	35.7 ± 4.3^a^	74.6 ± 9.0^a^	30.8 ± 0.4^b^	1658 ± 74^a^	14.3 ± 0.2^a^
	Oochikara	701 ± 26^ab^	(876)	25.1 ± 1.8^a^	73.3 ± 4.8^a^	39.7 ± 0.8^a^	1748 ± 87^a^	16.3 ± 1.7^a^
	Akita 39	625 ± 36^b^	(781)	33.1 ± 3.0^a^	83.9 ± 5.9^a^	23.2 ± 0.3^c^	1615 ± 56^a^	13.4 ± 1.0^a^
	Akitakomachi	554 ± 30^c^	(692)	28.8 ± 2.3^a^	85.8 ± 4.7^a^	22.9 ± 0.2^c^	1,441 ± 94^a^	11.9 ± 1.2^a^
0	Akita 63	627 ± 23^a^	(783)	23.7 ± 1.3^a^	88.9 ± 1.6^a^	30.4 ± 0.3^b^	1,260 ± 45^a^	10.1 ± 0.7^ab^
	Oochikara	511 ± 17^b^	(638)	16.4 ± 0.6^b^	79.3 ± 2.0^b^	41.1 ± 0.3^a^	1,343 ± 68^a^	12.1 ± 0.2^a^
	Akita 39	502 ± 30^b^	(628)	24.9 ± 1.1^a^	89.4 ± 2.3^a^	22.9 ± 0.4^c^	1,254 ± 54^a^	8.8 ± 0.7^bc^
	Akitakomachi	392 ± 28^c^	(489)	19.1 ± 1.0^b^	88.1 ± 2.0^a^	22.8 ± 0.2^c^	979 ± 40^b^	7.4 ± 0.5^c^

All cultivars were grown in in an experimental field of the Agricultural Experimental Station of Akita Prefecture in 2009, 2011, 2012 and 2013. Data are means ± SE of 3- and 4-year independent experiments. Different letters denote significant difference based on Tukey’s honestly significant difference test (α = 0.05). The value in parentheses indicates yield data shown as rough rice grain yield (international yield unit). Fertility indicates the ratio of filled grains to total grains.

### Yield and yield components of Akita 63 and the reference cultivars

Table 1 shows that the average brown rice yields of Akita 63 were 928 g m^−2^ (corresponding to 1,160 g m^−2^ of rough rice yield) at an application of 13 g N m^−2^, 780 g m^−2^ at an application of 6 g N m^−2^ and 624 g m^−2^ at an application of 0 g N m^−2^, respectively, for 4 years between 2009, 2011, 2012 and 2013. The yield level at an application of 13 g N m^−2^ was 1.7-fold higher than Japan’s average yield (Table S1) and 2.5-fold higher than the world average yield in 2018 (FAOSTAT (https://faostat3.fao.org). The yield levels of Akita 63 were about 20% higher than those of the parental lines Oochikara and Akita 39, and also 40–60% higher than those of the reference cultivar, Akitakomachi for the same N application. Whereas the single grain weight of Oochikara was the largest (40 mg per brown grain), the yield did not differ from that of Akita 39. For each N application, Oochikara tended to show greater biomass and higher total crop-N content. However, the number of grains of Oochikara was the smallest. Although there was no significant difference in the number of grains at an application of 6 g N m^−2^, this was offset by annual range (Supplementary Table S2). The number of grains of Oochikara was always the smallest at the same N application for all years. The ratio of filled grains to total grains (fertility) did not differ among cultivars except that the ratio in Akita 63 tended to be lower at high N application.

### Relationships between yield, yield components, biomass, and crop-N content

Wada and Matsushima^20^ reported that the amount of N uptake by rice plants determined the number of grains per unit of land area, irrespective of cultivars and paddy-fields. Therefore, we analyzed yield component parameters against total crop-N content of the above-ground section at harvest in four cultivars (Fig. 2A–D). While the number of grains did not differ among three cultivars including a large-grain cultivar, Akita 63, another large-grain cultivar, Oochikara, showed a lower number of grains at any given crop-N content (Fig. 2A, Supplementary Tables S3, Table S4). Total aboveground biomass at a given crop-N content did not differ among all cultivars, including Oochikara (Fig. 2B). Although the number of grains was linearly correlated with crop-N content passing through the origin, total biomass was curvilinearly correlated (*r* = 0.947 for the quadratic regression; *r* = 0.858 for the linear regression). The single grain weight was the greatest in Oochikara and remained constant at 40 mg, irrespective of crop-N content (Fig. 2C; Table 1). The single grain weight of Akita 63 remained at 30 mg and those of Akita 39 and Akitakomachi were both 22 mg. Since the single grain weight of Akita 63 was 35% larger and such a large grain weight was not associated with a decline in the number of grains, the yield was significantly higher for a given crop-N content (Fig. 2D, Supplementary Table S3, S4). On the other hand, although the single grain weight of Oochikara was 80% larger, this enlarged grain size was offset by an 80% decrease in the number of grains. Consequently, the yield of Oochikara did not differ from that of two other cultivars, Akita 39 and Akitakomachi. Thus, whereas a trade-off between single grain weight and number of grains was found for Oochikara, Akita 63 did not show such a relationship. The high-yielding potential of Akita 63 was due to overcoming a trade-off between enlarged grain size and reduction of the number of grains.

### Identification of genes determining grain size

**Figure 3:**
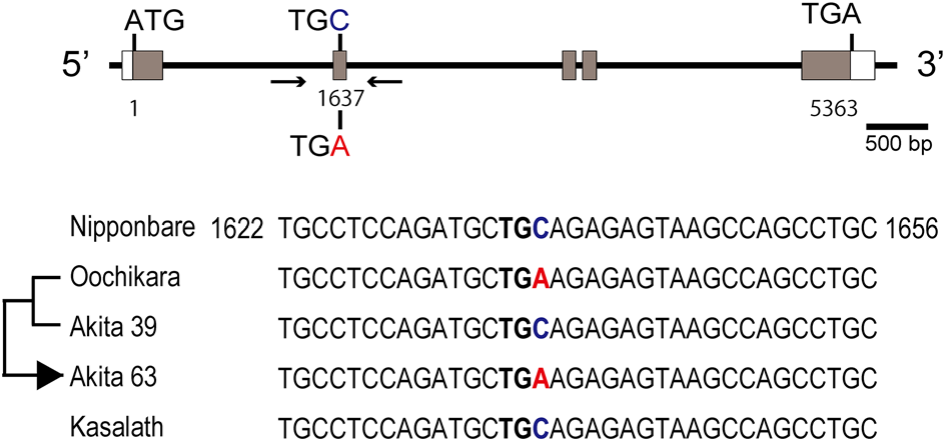
Mutation sites of *GS3* gene. The positions of coding regions (gray boxes), 5′ and 3′ UTRs (open boxes), translation start (ATG) and translation stop codons (TGA) are indicated. One common single nucleotide mutation at 1637-nucleotide in the second exon (Fan et al. ^11^) is indicated, in which a substitution of C (Nipponbare, Akita 39 and Kasalath) by A (Oochikara and Akita 63) in the second exon results in an early stop codon in Oochikara and Akita 63. Nucleotide sequences between 1622- and 1656-nucleotide of the *GS3* genes from Nipponbare, Akita 39, Oochikara, Akita 63 and Kasalath are displayed. Arrows indicate locations of primers that were used for PCR (Supplementary Table S6).

We cloned and sequenced several key genes determining grain size such as *GS3*, *GW2*, *TGW6* and *qSW5*. For all these genes, loss of function causes enlarged grains. *GS3* was isolated as the first grain-length QTL in different genetic backgrounds and identified as an unknown putative transmembrane protein by Fan et al.^11^. *GS3* gene has five exons with a transcript length of 956 bp encoding 232 amino acids, and one common single nucleotide mutation, a substitution of C by A, was found in the second exon at 1,637 nucleotide. This substitution results in a 178-aa truncation in the C-terminus of the predicted GS3 protein. Such a C to A mutation is widespread in the long-grained cultivars^21,22^, and this was also observed in the genomes of Oochikara and Akita 63 (Fig. 3). *GW2* as a major grain-width QTL encodes an unknown RING-type E3 ubiquitin ligase and a 1-bp deletion in the 4th exon causes premature truncation of the GW2 protein in a *japonica* cultivar, WY3, with large grains^12^. This mutation was not found in any of the cultivars examined in the present work, including Oochikara and Akita 63 (Supplementary Fig. S1). *TGW6* encodes IAA-glucose hydrase and contains a 1-bp deletion at 313-nucleotide in a long-grained *indica* cultivar, Kasalath^13^. This deletion was not observed in any of the cultivars examined in the present work (Supplementary Fig. S2). *qSW5* was isolated as a QTL for seed width in an *indica* cultivar, Kasalath^14^. A 1,212-bp deletion in *qSW5* leads to enlarged grain width. This deletion was observed in Oochikara, Akita 39 and Akita 63 in contrast to the allele of Kasalath (Supplementary Fig. S3). Thus, both Akita 63 and Oochikara have the large-grain alleles of *GS3* and *qSW5*.

## Discussion

Cereal crop yield is determined by three yield components, namely, the number of grains per unit of land area, grain weight, and the ratio of filled grains. In rice, single grain weight is genetically constant, irrespective of growth environments^5^. This character of rice is largely different from that of other cereal crops. For example, in wheat, single grain weight varies depending on growth conditions^23,24^, and a negative correlation is frequently observed between grain weight and number^23^. Therefore, in rice, an important target for achieving a high yield is to increase the number of grains with a high ratio of ripened grains. At the same time, this means that genetic enlargement of grain size has another great impact on increase in yields in rice. However, the relationship between grain size and yield has remained uncertain. Meanwhile, we found that a large-grain cultivar, Akita 63, exhibited a high yield (983 g m^−2^ of brown rice yield = 1,230 g m^−2^ of rough rice)^10^. The single grain weight of Akita 63 was 35% larger and the yield was 20–60% higher than that of the reference cultivars. According to our analysis, in spite of the large grain, the number of grains of Akita 63 did not differ from the common *japonica* cultivars at any crop-N content^10,25,26^. Therefore, a large grain size without reduction of the number of grains directly enhances the sink capacity, leading to high yield potential. However, although Oochikara, the maternal cultivar of Akita 63, has 80% larger grains, the yield was not necessarily high (560 g m^−2^ of brown rice yield^9,27^). The results in Fig. 2 clearly show that a large grain in Oochikara is associated with reduction of the number of grains and consequently, Oochikara has the same yield as that of reference cultivars with normal grains.

Among the fine-mapped major genes determining grain size, we examined the large-grain alleles of *GS3*, *GW2*, *TGW6* and *qSW5* in the present work. The results show that Akita 63 and Oochikara have the large-grain alleles of *GS3* and *qSW5* (Fig. 3; Supplementary Fig. S3). Lu et al.^28^ surveyed natural variation and artificial selection in major genes determining grain size among 127 varieties of rice cultivars, and reported that *GS3* and *qSW5* are major genes controlling grain size and that *japonica* cultivars with a nonfunctional *GS3* and *qSW5* genotype combination show the largest grain weight. Regarding this point, our results clearly coincide with their conclusion. However, as *qSW5* with a 1,212-bp deletion was also found for Akita 39 with normal grains, the effects of this allele on single grain weight may be limited. Actually, functional *qSW5* mainly originated from *indica* cultivars and leads to enlarged grain-length. The *qSW5* with a 1,212-bp deletion mainly originated from *japonica* cultivars and has an effect on the enlargement of grain width^14,29^.

The single grain weight of Oochikara is appreciably greater than that of Akita 63 (Table 1; Fig. 2C). Nevertheless, we did not find a difference in the large-grain alleles between them in our investigation. This indicates that Oochikara has other genes/alleles contributing to large grain size. At the same time, our results also indicate the possibility that Oochikara has other genes/alleles which function as a negative regulator(s) of the number of grains or no genes/alleles which function as a positive regulator(s). Although it is not known whether a trade-off between single grain weight and the number of grains in Oochikara is determined by the same gene(s), the breeding from Oochikara to Akita 63 overcomes such a trade-off trait. This means that the large-grain allele of *GS3* and *qSW5* combination does not affect the number of grains and can be one of major determinants for a further increase in yield. These two genes are widely observed in *Oryza sativa* species^14,21,28^, and *GS3* has stronger effects on grain weight in *japonica* cultivars^28^. Thus, although there still remains a possibility that other unidentified genes also come into play, it is suggested that the nonfunctional *GS3* and *qSW5* combination mainly contributes to the large grain size of Akita 63.

The 4-year average yields of Akita 63 were about 20% higher than those of the parents Oochikara and Akita 39, and 40–60% higher than those of the reference cultivar, Akitakomachi (Table 1). These results indicate that when Akita 63 was compared with Akitakomachi, the large grain of Akita 63 is not the sole determinant for high yield. Another factor was N uptake capacity. For the same N application, total crop-N content at the harvest stage tended to be higher in Akita 63 than in Akitakomachi (Table 1). Actually a significant difference in crop N content between them was found at an application of 0 g and 6 g N m^−2^ (Supplementary Table S2). This indicates that Akita 63 has superior N uptake capacity. This trait may have been inherited from the parental lines, Oochikara and Akita 39.

As already discussed above, to achieve a high yield, it is important to enhance the number of grains with a high ratio of ripened grains. In many cases, however, a negative correlation between the number of grains and the ratio of filled grains is frequently observed, especially when rice is cultivated with heavy N application^30^. This trend was clearly found for our data in Table 1. The ratio of filled grains to total grains decreased with increasing N application in all cultivars. Among them, the ratio of filled grains of Akita 63 was the lowest at an application of 13 g N m^−2^ for 3 years (Supplementary Table S2). As we previously pointed out, we think that this is caused by source limitation relative to yield potential^26^. Although the number of grains was linearly correlated with crop-N content passing through the origin, total biomass was curvilinealy correlated (Fig. 2A,B). Of course, the curvilinear correlation between biomass and crop N content was simply caused by a decline in canopy photosynthesis due to an excessive leaf area that may cause mutual shading at high N application. Grain mass (rough rice) in Akita 63 reached 60% of the total aboveground biomass at harvest, while that of other varieties reached about 45% (Table 1; Fig. 2B,D). This is the highest level of all cereal crops^31,32^. Thus, yield potential of high-yielding cultivars such as Akita 63 may surpass source capacity, leading to a decline in the ratio of filled grain. This indicates that a further increase in sink capacity is no longer effective and that improvements in source capacity will be essential for maintenance of high ratio of filled grains.

Many recent trials conducted at free-air CO_2_ enrichment (FACE) facilities have shown a highly positive correlation between enhanced photosynthesis, biomass and yield^32,33^. These results indicate that enhancement of photosynthesis by elevated [CO_2_] directly leads to an increase in yield when genetic factors besides photosynthesis are not altered^32^. Therefore, to examine the effects of source enhancement on yield, we conducted FACE experiments on several rice cultivars, including Akita 63^34^^.^ The results showed that Akita 63 had the greatest enhancement of yield by CO_2_ enrichment among all rice cultivars grown at FACE facilities. Furthermore, the absolute yield of Akita 63 was also highest and the ratio of filled grains remained at higher level. These results indicate that enhancement of photosynthesis is of the greatest importance for a further increase in the yield of high-yielding cultivars with a large sink size. While there has been a dispute as to whether photosynthesis improvement leads to an increase in cereal crop yields^35^, we have actually shown that an increase in photosynthesis by overproducing Rubisco results in increased rice yields under field conditions^36,37^ Thus, improving photosynthesis is a possible target for realizing a further increase in yield of today’s high-yielding cultivars.

## Conclusions

The world’s population is now exponentially increasing and is predicted to reach 10 billion by the middle of this century. An increase in the population up to now has been partly sustained by an increase in food supplies due to the success of the dwarfing of rice and wheat with large inputs of N fertilizer and herbicides^4^. In order to feed a population of 10 billion within the next 30 years, however, a further increase in yield beyond the innovations of the Green Revolution will be required. Our studies with a large-grain cultivar, Akita 63, indicate that enlargement of single grain size can have a great impact on further increases in yield potential. In addition, Akita 63 has superior N uptake capacity and shows higher physiological N use efficiency. At the same time, our present studies demonstrate that further improvement of sink capacity is limited for today’s high-yielding cultivars, as exemplified by Akita 63 because source limitation occurs relative to high yield potential. Both sink and source improvements in main crops will be essential for the Second Green Revolution.

## Materials and methods

### Plant culture

A large-grain rice (*Oryza sativa* L. cv Akita 63) was grown at different levels of N application in an experimental field of the Agricultural Experimental Station of Akita Prefecture, Oogata-mura, Akita, Japan (40° 0′ N, 140°0′ E, − 3.7 m altitude) in 2009, 2011, 2012 and 2013. As reference *japonica* rice cultivars, a maternal cultivar, Oochikara, which was bred as a large grain cultivar^9,27^, a paternal cultivar, Akita 39, and a conventional cultivar, Akitakomachi, which is the present leading cultivar in Akita Prefecture and known as one of the most tasty types of rice in Japan were grown under the same conditions. All cultivars except Oochikara were bred at this Agricultural Experimental Station, and Oochikara was bred at NARO Agricultural Research Center, Niigata, Japan.

Seedlings were grown in a plastic greenhouse from the beginning to the middle of April until about the middle of May, and were then transplanted at a hill spacing of 0.3 m × 0.16 m (21 hills m^−2^) with four to five seedlings in an experimental paddy field. Soil type was Heavy Clay soil (Eutric Fluvisols ; FAO) with pH 6.7, 23.8 g/total C kg, 2.4 g/total N kg and 32.3 cmol/kg cation exchange capacity. Plants were grown at three levels of N fertilization: 0, 6, and 13 g N m^−2^. For the standard level of N application, 4 g N m^−2^ of (NH_4_)_2_SO_4_ was used as a basal fertilizer, and 2 g N m^−2^ of (NH_4_)_2_SO_4_ was used as a top dressing fertilizer at the young panicle formation stage (6 g N m^−2^ in total). For zero-N application, no N fertilizer was used. For a high level of N application, 4 g N m^−2^ of (NH_4_)_2_SO_4_ and 7 g N m^−2^ of LP100 (polyolefin-coated urea, Chisso Co., Japan) were used as basal fertilizers and 2 g N m^−2^ of (NH_4_)_2_SO_4_ was used as a top dressing fertilizers at the young panicle formation stage (13 g N m^−2^ in total). Phosphorus (4 g P m^−2^) and potassium (4 g K m^−2^) were applied to both N application plots before plowing. The size of each plot was 28.8 m^2^ (3.6 m wide and 8 m long). All cultivars were heading (the time when 80% of the panicles had emerged) from August 8 to 17, and were harvested at the end of September when more than 90% of the grains had turned yellow. Panicle numbers were examined for 20 hills of each plot, and three hills with a mean panicle number from each plot were collected. Plants were separated into leaf blades, culms plus sheaths and panicles. These samples were oven-dried at 85–100 °C for more than 1 week, and weighed. At the same time, three additional hills with a mean panicle number were harvested and used for measurement of the ratio of filled grains to total grains. This ratio was determined by submerging the grains in an NaCl solution with a specific gravity of 1.06. The filled grains were then hulled and oven-dried at 85 to 100 °C for the determination of grain dry weight. A survey of yield was carried out for 80 hills from the center part of each plot. Rough rice grains from 80 hills were hulled and put through a 1.8 mm sieve to remove immature kernels. The hulled rice grains (brown rice grains) were also oven-dried at 85–100 °C for more than 1 week and weighed. The final brown rice yield was corrected at a moisture content of 0.14 g H_2_O g^−1^ fresh weight. When the yield was expressed as rough (unhulled) rice yield, it was multiplied by a conversion factor of 1.25^5^.

For determination of total plant N, dried materials were powdered and then N was determined with an N analyzer (Rapid N Cube, Elementar Analysensysteme GmbH, Frankfurt, Germany).

During these experimental years (2009, 2011, 2012 and 2013), the average yields of rice in Akita prefecture and Japan and the climate conditions at Oogata-mura, Akita, Japan are shown in Supplementary Tables S1 and S5, respectively.

### Genes determining grain size

*GS3*, *GW2*, *TGW6* and *qSW5* (AB488612, AK065504, AB513135 and AB433345 in Gene Bank; https://www.ncbi.nlm.nih.gov/genbank/) were sequenced in the fresh leaves of rice seedlings including a *japonica* cultivar Nipponbare and an *indica* cultivar Kasalath according to our previous protocol^38^. Genomic DNA in leaves was extracted according to Obara et al.^39^. Gene fragments were amplified from genomic DNA using rTaq (TaKaRa Biotechnology, Japan) using specific primer pairs designed according to the corresponding gene sequences deposited at RAP-DB (The Rice Annotation Project; https://rapdb.dna.affrc.go.jp/index.html) and MSU (Rice Genome Annotation Project; https://rice.plantbiology.msu.edu/index.shtml). The gene loci and primer sequences codes are described in Table S4. The amplified PCR products were cloned into PCR4-TOPO (Technologies Corporation), four to five independent plasmid DNAs were randomly selected, respectively, and then fully sequenced. The DNA sequences were aligned using the ClustaIW program (https://clustalw.ddbj.nig.ac.jp/) and manually adjusted in GENETYX Ver.10 (GENETYX CORPORATION, Japan).

### Statistical analysis

Data are presented as the mean ± standard error. Tukey–Kramer’s honestly significant difference test was performed with JMP (SAS Institute Inc., Cary, NC, USA). Scatter diagrams, regression lines, Pearson’s product moment correlation coefficients (*r* value) using Excel (Microsoft) and the correlation significance identified by Spearman’s rank-order correlation (*P* value) were created and calculated using Excel Tokei (BellCurve, Social Survey Research Information Co., SAS Institute Inc.). Covariance analysis was conducted using Excel Tokei (BellCurve), and significance levels were corrected using the Bonferroni method to avoid family-wise error (significance level/test number) when statistical tests were repeated.

## Supplementary information

Supplementary information

## Data Availability

The data used or analyzed during this study are available from the corresponding author on reasonable request.
