# Supplementary material for: High yielding ability of a large-grain rice cultivar, Akita 63
Source: Sci Rep. 2020 Jul 22;10:12231. doi: 10.1038/s41598-020-69289-0 (PMC7376063; doi:10.1038/s41598-020-69289-0)
Supplement: Supplementary file 1 — Supplementary information [file 41598_2020_69289_MOESM1_ESM.pdf]

High Yielding Ability of a Large-grain Rice Cultivar, Akita 63

Amane Makino, Yoshihiro Kaneta, Mitsuhiro Obara, Keiki Ishiyama, Keiichi Kanno, Eri Kondo, Yuji Suzuki and Tadahiko Mae

Table S1. Mean values of yield (brown rice) and yield components of rice in Akita prefecture and all Japan during the experimental years[a].

	2009				2011				2012				2013			
	Yield[a]	Grn No[b]	Fertility[c]	Grn wt	Yield	Grn No	Fertility	Grn wt	Yield	Grn No	Fertility	Grn wt	Yield	Grn No	Fertility	Grn wt
	$g\ m^{-2}$	*$10^3\ m^{-2}$*	*%*	*mg*	*$g\ m^{-2}$*	*$10^3\ m^{-2}$*	*%*	*mg*	*$g\ m^{-2}$*	*$10^3\ m^{-2}$*	*%*	*mg*	*$g\ m^{-2}$*	*$10^3\ m^{-2}$*	*%*	*mg*
Akita	579	31.4	87.2	21.1	581	31.2	85.5	21.8	585	30.4	89.5	21.5	585	31.0	87.0	21.7
	(99)[c]	(99)	(101)	(99)	(99)	(98)	(100)	(102)	(100)	(96)	(102)	(100)	(100)	(97)	(101)	(101)
All Japan	535	29.5	84.4	21.5	549	29.8	84.3	21.8	551	29.5	86.5	21.6	551	30.1	85.0	21.5
	(99)	(99)	(99)	(100)	(101)	(100)	(99)	(101)	(101)	(99)	(101)	(100)	(101)	(102)	(100)	(100)

[a] Data are published at the website of the Ministry of Agriculture, Forestry and Fisheries, Japan (in Japanese) (http://www.maff.go.jp/j/tokei/kouhyou/sakumotu/sakkyou_kome/index.html).

[b] Grn No; number of grains

[c] Fertility; the ratio of filled grains to total grains

[d] The value in parentheses indicates that 100 corresponds to the average on each component during the latest 10 years.

Table S2. Yield (brown rice) and yield components, total N content of plant aboveground at harvest of Akita 63, Oochikara, Akita 39, and Akitakomachi in each year from 2009 to 2013

	2009					2011					2012					2013				
	Yield[a]	Grn No[b]	Fertility[c]	Mass[d]	Total N	Yield	Grn No	Fertility	Mass	Total N	Yield	Grn No	Fertility	Mass	Total N	Yield	Grn No	Fertility	Mass	Total N
	g m^{-2}	*10^{3}m^{-2}*	*%*	*g m^{-2}*	*g m^{-2}*	*g m^{-2}*	*10^{3}m^{-2}*	*%*	*g m^{-2}*	*g m^{-2}*	*g m^{-2}*	*10^{3}m^{-2}*	*%*	*g m^{-2}*	*g m^{-2}*	*g m^{-2}*	*10^{3}m^{-2}*	*%*	*g m^{-2}*	*g m^{-2}*
13 N g m^{-2}																				
Akita 63	887	40.0	73.0	1786	17.3	868	52.9	53.9	1911	18.3	1003	46.8	74.0	2001	19.8	955	45.7	74.9	2078	ND[e]
Oochikara	704	24.1	77.0	1943	21.1	692	29.4	60.6	1881	20.2	849	31.3	80.6	2150	21.6	919	29.5	77.1	2416	ND
Akita 39	707	42.7	75.0	1590	18.8	733	62.9	50.9	2150	22.4	779	41.9	86.7	1986	18.1	859	43.2	87.8	1835	ND
Akitakomachi	606	35.3	80.0	1582	17.6	603	42.7	62.1	2016	20.1	621	33.8	84.9	1542	16.2	711	38.3	84.0	1778	ND
6 N g m^{-2}																				
Akita 63	726	33.5	74.0	1600	14.0	751	48.3	49.2	1476	14.7	756	28.5	87.8	1790	14.1	887	32.6	87.3	1764	ND
Oochikara	689	21.8	78.0	1606	13.9	672	29.7	59.5	2002	19.5	667	22.8	81.4	1686	15.4	777	26.1	74.2	1697	ND
Akita 39	634	36.1	81.0	1567	13.4	652	39.4	68.7	1710	15.2	523	25.7	90.1	1479	11.7	692	31.3	95.7	1703	ND
Akitakomachi	593	33.9	81.5	1469	13.7	527	30.1	75.0	1352	12.2	483	23.4	90.8	1253	9.7	612	27.7	95.8	1689	ND
0 N g m^{-2}																				
Akita 63	655	26.7	86.0	1134	9.7	658	24.9	86.8	1309	11.3	558	21.1	89.6	1260	9.1	635	22.0	93.3	1338	ND
Oochikara	527	16.0	79.0	1221	12.4	466	15.0	76.5	1480	12.3	505	17.6	85.1	1442	11.6	544	16.9	76.6	1231	ND
Akita 39	483	24.3	84.0	1254	8.3	560	26.4	89.5	1349	10.2	425	21.9	88.7	1161	8.0	541	26.9	95.3	1253	ND
Akitakomachi	410	19.2	85.0	1024	7.4	377	18.2	89.6	938	8.2	323	17.0	84.7	888	6.6	456	21.8	92.9	1066	ND

[a] Yield data are shown as brown rice yield.

[b] Grn No; number of grains

[c] Fertility; the ratio of filled grains to total grains

[d] Mass; total aboveground biomass

[e] ND: not determined.

Table S3

Correlation between grain number, total biomass, brown rice yield and total crop-N content of plant aboveground of Akita 63, Oochikara, Akita 39 and Oochikara

	Cultivar	Sample size	Regression line	*r*	*P*	
**Grain number**	Akita 63	21	y = 2570x - 1490	0.844	0.000	**
	Oochikara	23	y = 1440x - 280	0.898	0.000	**
	Akita 39	22	y = 2610x - 1240	0.947	0.000	**
	Akitakomachi	20	y = 1930x + 4480	0.957	0.000	**
**Brown rice yield**	Akita 63	22	y = 35x + 265	0.929	0.000	**
	Oochikara	23	y = 24x + 244	0.726	0.000	**
	Akita 39	22	y = 22x + 294	0.884	0.000	**
	Akitakomachi	23	y = 20x + 237	0.867	0.000	**

Regression lines and Pearson's product moment correlation coefficients (*r*) are shown between each parameter and plant N content of the above-ground. The significance of correlations, identified by Spearman's rank-order correlation coefficient (*P*), is marked by asterisks; * indicates $P<0.05$, ** indicates $P < 0.001$; "n.s" indicates no significance.

Table S4

Significant difference test between the slope and y-intercept of the regression line between the two cultivars

	Fixed factor	Slope (*P*)		y-intercept (*P*)		Valuation
**Grain number**	Akita63 vs Oochikara	0.006	n.s	0.000	**	Parallel line
	Akita 63 vs Akita 39	0.928	n.s.	0.634	n.s.	Same line
	Akita 63 vs Akitakomachi	0.123	n.s	0.172	n.s	Same line
	Oochikara vs Akita 39	0.000	**	-	-	Intersect[a]
	Oochikara vs Akitakomachi	0.024	n.s	0.000	**	Parallel line
	Akita39 vs Akitakomachi	0.014	n.s	0.029	n.s	Same line
**Brown rice yield**	Akita63 vs Oochikara	0.103	n.s	0.000	**	Parallel line
	Akita 63 vs Akita 39	0.006	n.s.	0.000	**	Parallel line
	Akita 63 vs Akitakomachi	0.003	n.s	0.000	**	Parallel line
	Oochikara vs Akita 39	0.751	n.s.	0.351	n.s.	Same line
	Oochikara vs Akitakomachi	0.560	n.s	0.027	n.s	Same line
	Akita 39 vs Akitakomachi	0.706	n.s	0.000	**	Parallel line

Covariance analyses were conducted between each parameter and total crop-N content of the above-ground section of Akita 63, Oochikara, Akita 39 and Akitakomachi. Significant differences in the slope and y-axis intercept of two regression lines are summarized. Significance differences (*P*) obtained, correcting using the Bonferroni method to avoid familywise error (*P*-value/test number = 6), are marked with asterisks; * indicates $P < 0.0017$; ** indicates $P < 0.00017$. "n.s" indicates no significance. [a] Coordinate point of intersection for two regression lines between Oochikara and Akita 39 is (1.07 g N m^{-2}, 1260 m^{-2}), but there is no this coordinate point in the x-axis effective range (8.9 to 22.1 g N m^{-2}) (See; Fig 2).

Table S5. Climate conditions at the experimental paddy field in each year from 2009 to 2013

Year		May			June			July			Aug.			Sep.			Oct.		
		Big	Mid.	End	Big.	Mid.	End	Big.	Mid.	End	Big.	Mid.	End	Big.	Mid.	End	Big.	Mid.	End
Av.	Temp. (°C)	12.3	13.5	15.0	16.9	18.2	19.4	20.5	21.8	23.4	24.1	24.0	23.0	21.4	19.3	17.3	15.3	13.3	11.2
	Sunshine (h)	6.0	5.8	6.5	6.4	5.8	5.2	4.8	4.9	5.8	6.2	6.5	6.2	5.4	4.9	4.9	4.7	4.7	4.1
2009	Temp. (°C)	15.1	13.4	15.4	17.2	17.3	22.6	21.2	21.8	22.4	23.9	23.9	21.2	20.3	17.7	18.1	15.3	14.4	12.9
	Sunshine (h)	8.6	5.8	5.2	2.7	6.5	6.8	3.9	2.2	3.1	4.1	5.9	5.9	6.2	6.7	4.4	4.8	5.2	5.6
2011	Temp. (°C)	11.4	14	14.7	17.3	18.1	19.9	23.1	25.7	23.0	25.7	25.8	22.4	24.6	21.1	16.7	14.1	14.5	13.4
	Sunshine (h)	4.4	6.0	5.3	6.7	7.1	2.3	3.7	8.6	5.3	9.4	5.3	5.4	3.9	2.8	6.6	3.9	5.1	4.1
2012	Temp. (°C)	14.7	12.6	15.9	18.2	19	18.9	22.1	21.8	24.6	24.9	25.1	27.2	25.6	25.2	19.2	17.3	15.2	12.9
	Sunshine (h)	3.9	4.6	6.1	7.9	7.1	8.8	4.8	5.8	5.8	8.0	6.5	9.5	7.0	7.5	4.2	6.4	5.5	4.2
2013	Temp. (°C)	10.0	13.9	17.0	18.0	21.9	20.5	23.7	21.9	23.9	24.9	26.6	23.3	21.2	21.1	18.7	18.7	13.7	13.3
	Sunshine (h)	3.8	3.1	6.4	9.9	6.8	7.9	3.2	4.8	2.0	4.6	5.7	6.0	3.5	5.5	7.0	4.3	4.5	1.7

Climatic conditions at Oogata-mura where Agricultural Experimental Station of Akita Prefecture locates (40˚0'N, 140˚0'E, -3.7 m altitude) from May to October in 2009, 2011, 2012 and 2013. This covers the rice cultivation period in the experimental paddy field. For "Temp.," yellow and light blue highlights indicate a change in the average temperature of >1.0 °C or <–1.0 °C, respectively. For "Sunshine," orange and light blue highlights indicate a period of time with >120% or <80% of the average sunshine duration, respectively. Gray highlights indicate a temperature within 1 °C of the average temperature and a sunshine duration that was 80%–120% of the average. Data on the average weather over the past 30 years at Oogata-mura are available on the Japan Meteorological Agency (JMA) website (JMA, http://www.data.jma.go.jp/gmd/risk/obsdl/index.php).

Table S6. List of primer sequences for gene analyses.

Gene	RAP-DB code	RGAP code	Accession No.		Sequence
GS3	Os03g0407400	-	AB488612	Forward	5'- GACAGTACTTGCTGTCTAGCTTT -3'
				Reverse	5'-AAGTAAGTATATTTTGTGAATGCAT -3'
GW2	Os02g0244100	LOC_Os02g14720	AK065504	Forward	5'- AACTGTTTGACAACCACTCC -3'
				Reverse	5'- TATGTTAGTTGCAGCCTGTG -3'
TGW6	Os06g0623700	LOC_Os06g41850	AB513135	Forward	5'- TCTCCGACGGCCGCATCATG -3'
				Reverse	5'- CGATGAAGCGCTATCCAATA -3'
qSW5	Os05g0187500	LOC_Os05g09520	AB433345	Forward	5'- ACCTCAGTTAACAGTTTATC -3'
				Reverse	5'- CGATAACCATCGGTAATTGCTA -3'

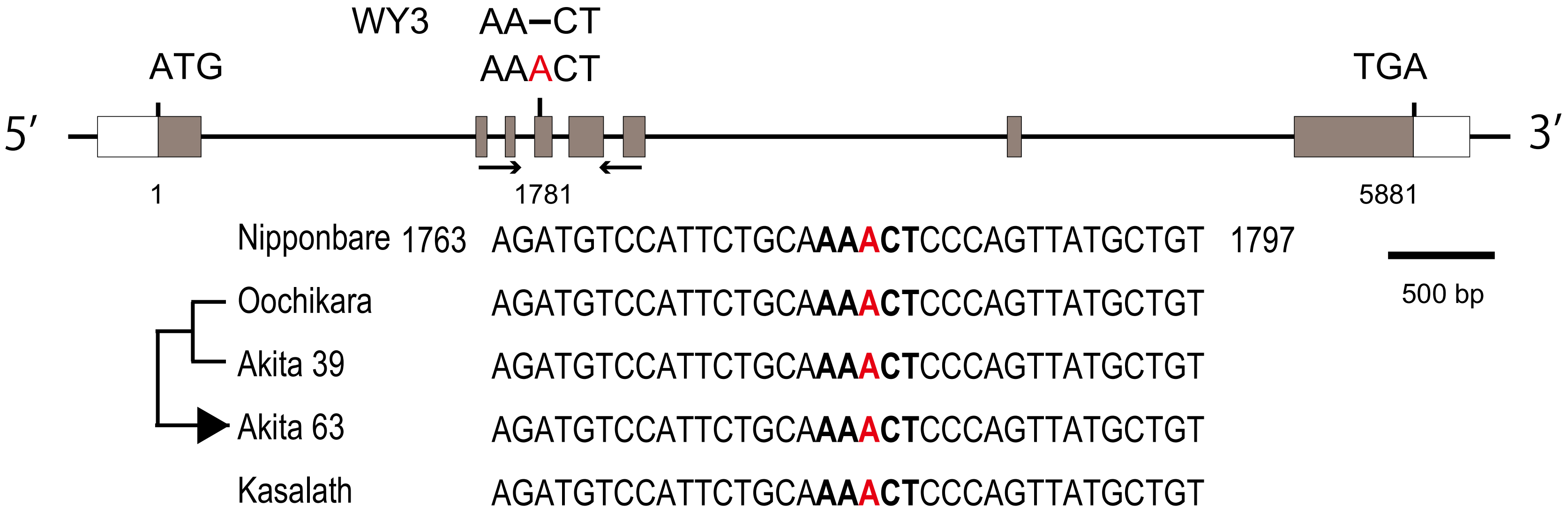

Figure S1. Mutation sites of *GW2* gene. The positions of coding regions (gray boxes), 5' and 3' UTRs (open boxes), translation start (ATG) and stop codons (TGA) are indicated. One common single nucleotide mutation at 1781-nucleotide in the fourth exon is indicated, in which with 1 bp-deletion of A in the fourth exon in a *japonica* cultivar, WY3, with large grains results in an early stop codon (Song et al. 2007). Nucleotide sequences between 1763- and 1797-nucleotide of the *GW2* genes from Nipponbare, Akita 39, Oochikara, Akita 63 and Kasalath are displayed. Arrows indicate locations of primers that were used for PCR (Supplemental, Table S3).

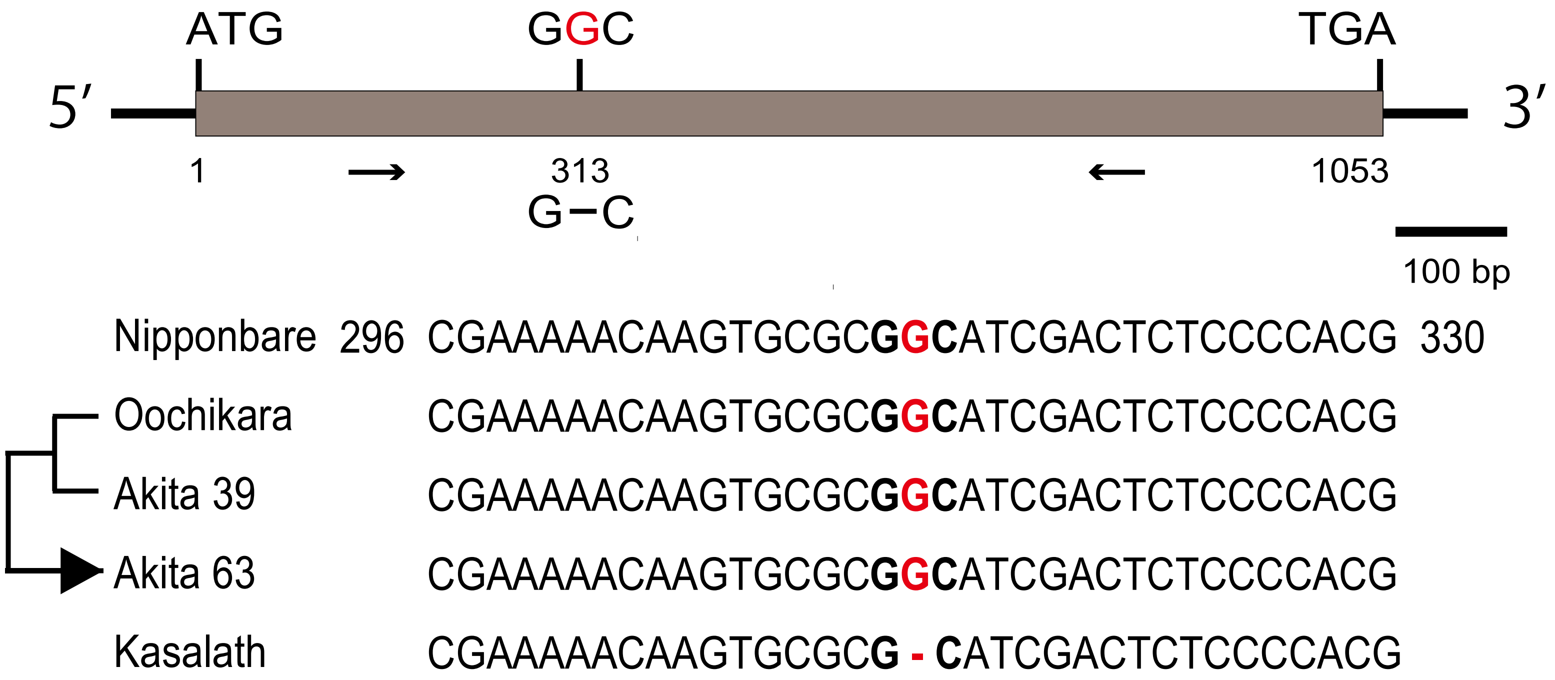

Figure S2. Mutation sites of *TGW6* gene. The positions of coding region (gray box), translation start (ATG) and stop codons (TGA) are indicated. The *TGW6* gene contains a single ORF with 1 bp-deletion of G at 313-nucleotide in an *indica* cultivar, Kasalath with long grains (Ishimaru et al. 2013), resulting in an early stop codon. Nucleotide sequences between 296- and 330-nucleotide of the *TGW6* from Nipponbare, Akita 39, Oochikara, Akita 63 and Kasalath are displayed. Arrows indicate locations of primers that were used for PCR (Supplemental Table S3).

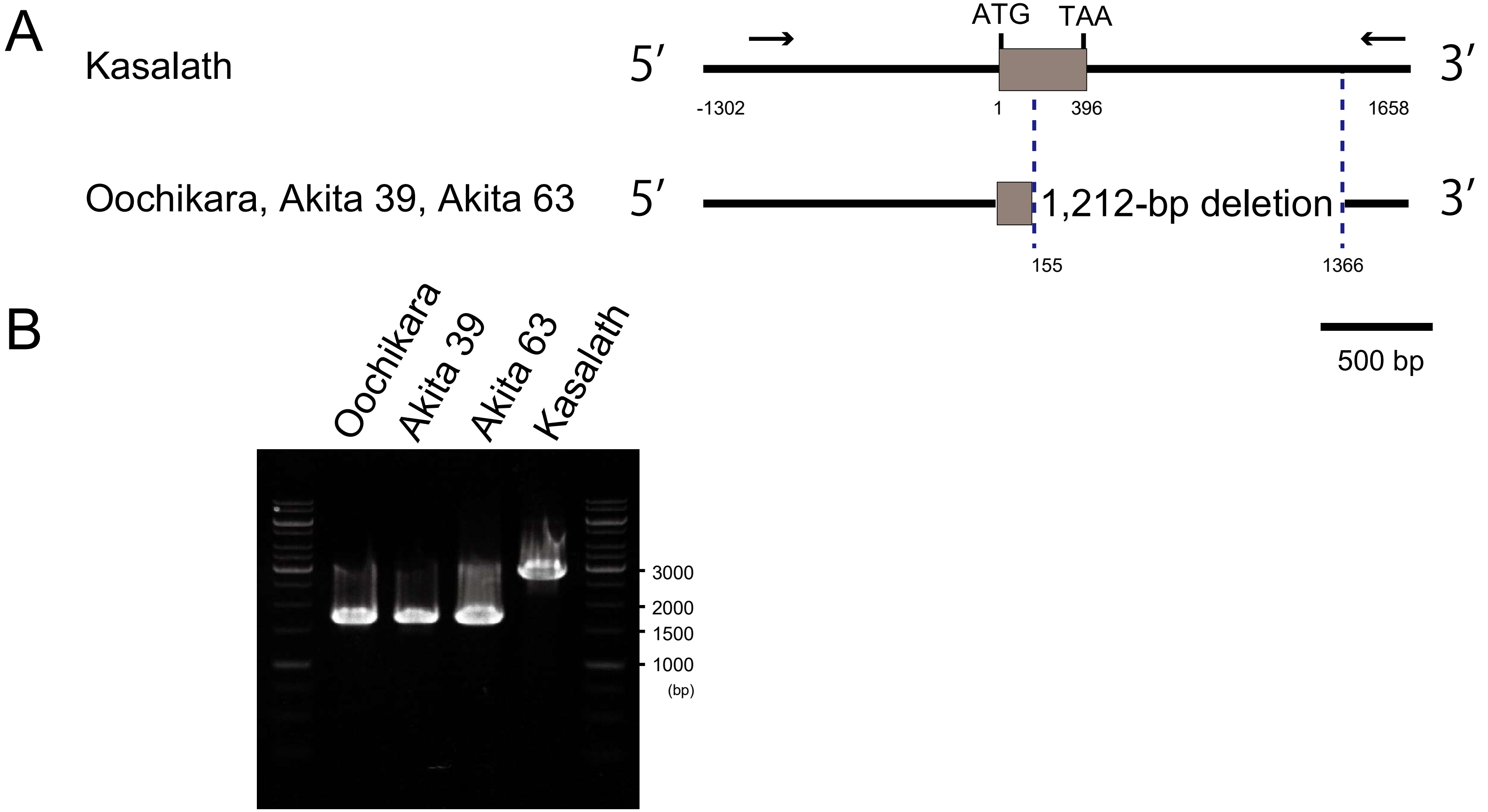

Figure S3. Mutation sites of *qSW5* gene. (A) and electrophoresis loaded on PCR products against the *qSW5* alleles in the four rice cultivars (B). The positions of coding regions (gray boxes), translation start (ATG) and stop codons (TGA) are indicated. In the *qSW5* alleles of Akita 39, Akita 63 and Oochikara, a 1,212-bp deletion from 155- to 1,366-nucleotide is found, in contrast to the allele of Kasalath. Arrows indicate locations of primers that were used for PCR (Supplemental Table S3).
